# Transactions of the Medical Society of the County of Kings

**Published:** 1864-11

**Authors:** 


BUFFALO

### Medical and Surgical Journal.

VOL. IV. NOVEMBER, 1864. No. 4.

ART. I.—*Transactions of the Medical Society of the County of Kings. Regular Meeting June*, 1862. *Medullary Cancer—Hydrothorax, with distinct tubal murmur—Paracentesis Thoraxsis—Death and post mortem*

*Dr. A. N. Bell* presented a tumor and its surroundings, taken from the anterior mediastinum of a patient that had recently died in the Brooklyn City Hospital. An Irish sailor, aged 21, of lymphatic temperament, entered the hospital on the evening of May 26th, and was first visited on the morning of next day. The patient was a few days from Fortress Monroe, whence he came in hospital ship, in consequence of "*sore thro at*," contracted as represented two weeks before by exposure to inclement weather. Previous to this he had no recollection of ever having been sick. He lay in a semi-recumbent position on his back, breathing with difficulty from evident obstructions. The cervical glands of both sides were much swollen close up to the trachea, and on the right side complicated with a swelling of the submaxillary gland, as large as a medium sized potatoe, and also extending to a swelling of the superficial glands of the parotid region, half as large as the patient's fist. The tonsils, too, were much swollen, of livid color, studded with numerous white specks. Respirations were forty per minute, frequently interrupted by a short cough and the expectoration of small quantities of frothy mucus streaked with blood. The right side of the thorax was motionless and two inches larger than the left, and the *feel* was characteristic of hydrothorax. Yet, on auscultation, *there was distinct tubal murmur over the whole extent of right lung.* On the left side there was puerile respiration, but apparently too little to compensate for the deficiency in the other lung, and deficient resonance over the upper lobe. His skin was hot and covered with profuse perspiration. Pulse 130, small and irritable. An oiled-silk jacket was applied and fifteen grains of chlorate af potash ordered to be taken every three hours. Beef tea and gruel, for diet.

The next day at twelve o'clock the medical staff was convened for consultation. Excepting that there was increased dypnœa, the patient's condition was pretty much the same. It was determined to tap the chest, and this operation Dr. Bell performed by making a valvular opening for the trocar through the eighth intercostal space of the right side, in a line perpendicular to the axilla. The pump was attached to the trocar and 137½ ounces of dark straw colored fluid drawn off. The patient bore the operation well, and on its conclusion coughed a good deal. Pulse fell to 84, and became moderately full. Respirations proportionately slow. A little stimulus was given, and he expressed himself as feeling comfortable. On the whole he appeared to be in such a good condition, that it was deemed best to leave him to future indications. At 4 o'clock P. M. there was considerable reaction, and his pulse rose again in frequency to 130, but he still expressed himself as comfortable, and enjoyed a cup of warm gruel. He continued to cough a good deal, and expectorated a large quantity of frothy mucus without difficulty. At 2 o'clock on the morning of the 29th he roused the nurse, who was near him, for water; sat up in bed and coughed hard for some minutes, again became quiet and went to sleep. Three and a half hours afterwards the attention of the nurse was attracted by his sudden struggles, following an effort to turn himself, in the agonies of death! Before the house physician could get to him he had suffocated.

*Post mortem* seven hours after death. On raising the sternum the specimen here presented—a mass of transformed mediastinal glands and abnormal growth, as large as a man's foot, filling the whole space from the sternum to the root of the lungs—was revealed. It was strongly adherent on all sides, pressing upon the trachea and its bifurcations. The structure of the tumor appeared to be irregularly lobulated. The whole mass was more or less infiltrated with pus, while there were numerous cavities resembling softened tubercle in the lung. The pleura between the tumor and the diaphragm, and of the right side, was three-eights of an inch thick, and strongly adherent to the pleura pulmonalis; thus forming a *solid connection from the root of the lungs to the walls of the chest, and accounting for the communicated tubal sound in auscultation with hydrothorax.* The pericardium was also adherent to the tumor. In the right pleural sac there remained about a pint and a half of serum. The lung was nearly solid, and rather less than one-fourth its natural volume. There were also some adhesions posteriorly, increasing the continuity of the connection on this side with the bronchi of the left. The left lung was free and ctepitant throughout, and the pleural sac healthy. The kidneys presented a knotty appearance, the surface being composed of rounded protuberances from one-quarter to a half an inch in diameter. The cortical portion conjested, and one-third thicker than natural.

The death of the patient was from *apnœa*, caused by the pressure of the mediastinal tumor on the trachea and its primary branches, and was not hastened by the paracentesis. Dr. Bell had carefully examined the specimen under the microscope, and some of his friends had also, but owing to the broken down condition of the tissue its *intimate* structure could not be ascertained. Yet he had no doubt from the appearance of the tumor that it was cancerous, and he believed it to be of the species denominated by pathologists, *medullary*, and of rapid growth.

*Albuminuria*—"*White Kidney*," *with cysts.*—Dr. Bell also presented the kidneys of a hospital patient sixty-two years old, who died of *Bright's disease.* The patient for three days previous to his death had secreted only four ounces of urine—and for the last twenty-four hours previous to death—none whatever. The specific gravity of the urine was 1008, while it deposited one-third of its volume of albumen. The kidneys were one-fourth larger than natural, were of pale color—and a good example of what is commonly known as "white kidney"—excepting that they contained several small cysts, a condition in *Bright's kidney* believed to be rare.

*Dr. Enos* enlarged upon the interest of the first specimen, presented by Dr. Bell, with special reference to its cancerous character, even though the microscope failed to reveal it. He thought it probable however, that the mediastinal tumor was of long duration.

### *Ulceration of the Bowels.*

*Dr. Ball* submitted a specimen of extensive ulceration of the bowels. The patient was a woman about 60 years of age, who had enjoyed good health, until a month previous to her last illness. Upon examination he discovered a tumor, as large as the fist, in the vicinity of the right ovary. There was considerable tenderness over the tumor, and also some gastric tenderness; yet upon the whole, there was scarcely any pain. He suspected ovarian disease. She finally sank and died two months after the beginning of illness. On post mortem the lower part of the colon was found to be engorged, and adherent to surrounding parts; the ileum was also indurated and ulcerated, and very much contracted. The appendix vermiformis was large and filled with fæces. He considered the case somewhat remarkable from its protracted character—with such severe lesion, accompanied by so little suffering. The right ovary contained a small, fibrous tumor.

### *Chloroform Poisoning.*

*Dr. G. K. Smith* read a paper on "Chloroform Poisoning," administered by the mouth. He observed that besides a case which had fallen under his own observation, he had collected several others, which went to show that chloroform, when taken as a poison, or administered in practice, was much less deadly than what is commonly believed.

The Doctor retained his paper with a view to further elaboration.*

*Dr. Bell* made some remarks on the sedative and anodyne effects of chloroform, and the amount necessary for a dose. In some cases he had been able to produce quietude with it when opium had failed, and in several instances he had given it in combination with opium, with decided benefit. He had especially used it with benefit, in incipient *mania-a-potu*, and frequently in colic, both alone, and with opiates or stimulants. He usually administered it mixed with glycerine, or syrup of gum arabic, in doses of from ten to fifteen drops every hour, till its effects were manifest. It was rarely necessary, however, to repeat the dose. In colic it was well to add a little acoholic stimulus.

*Dr. Otterson* elicited the opinion of Dr. Bell, that he would also unhesitatingly order it in lead colic, and would consider it an appropriate remedy, in doses to the extent of thirty drops without any danger of ill effects from its use.

*Dr. Ford* gave his experience in the use of chloroform as a sedative, in doses of fifteen drops, and compared its effects to chloric ether.

*Dr. Mason* thought it a commendable remedy, especially when combined with opium; that ten or fifteen drops, when added to a comparatively small quantity of morphine, very much increased the usual sedative effects of the salt.

### *Pneumonia in the Army of the Potomac.*

*Dr. Olcott* gave a brief recital of a few weeks experience at Fortress Monroe, observing, among other facts, that many patients died of *pneu-*

* Owing to Dr. Smith's absence his paper cannot be obtained for its appropriate place in the *Transactions.*

*monia*, consequent upon comparatively slight injuries and operations. This was not satisfactorily accounted for, but was generally supposed to depend upon pyæmia.

*Dr. Johnson* raised the inquiry as to the influence of malaria in the large mortality from wounds and operations, among the persons in the swamps of the Chickahominy.

*Dr. Bell* stated that he was familiar with the climatology of that section of the country, and he felt persuaded in his own mind that the great prevalence of pneumonia there under the circumstances was due to *malaria*, and net to *pus poisoning*. He also stated that the statistics of disease in the various sections of the country, as collected by Blodget, La Roche, and other authorities on the climatology of the United States, showed that pneumonia was common to various localities in the Southern States; and further, that according to his own observation, the region of country now under consideration was a locality of that kind. He had also observed congestive pneumonia following the autumnal remittent fever in that section, and that it was usually of short duration and very fatal.

*Cystic Disease of the Ovary.*

*Dr. Enos* exhibited a uterus and ovaries, removed from a subject in the dissecting room. There were two cysts connected with the right ovary, one of which, when distended, was about the size of a turkey's egg; the other was small and not opened. The cyst contained a thick, honey-like substance, of an oleaginous nature. It also contained a quantity of hair. There was no other morbid deposit. The cyst, he presumed, commenced in the graaffian vesicles, which were entirely absorbed by the progress of the disease.

---

*Quarterly Meeting, July* 8, 1862—*Annual Address by* SAMUEL HART M. D., *President of the Society.*

I rise, gentlemen, in compliance with a requirement of the by-laws of our Society, not expecting to present anything new, or worthy of special consideration, and must bespeak your indulgence to the few thoughts I shall suggest; they are but the transcript of views and opinions I long ago embraced, and which every succeeding year has served but to strengthen and confirm.

Observation and experience minutely analyzed and rigidly elaborated, are the only sure basis of accurate conclusions in scientific investigations, and that can lead to results which are conclusive and irresistible. An accumulation of facts, passing the ordeal of the severest scrutiny of many tests, and perhaps many ages, alone can establish a principle with demonstrative certainty. Science is but progressively developed; and the maxim of the ancients, "*veritas in putco*," every subsequent research has fully illustrated. Deep, reflecting minds have suggested thoughts, which have furnished data for succeeding inquirers, and these again to those of later times. Galileo produced a telescope, but was not this success acquired by hints thrown out by Roger Bacon, who made some advances in this direction two and a half centuries before? This memorable instrument has received great improvement since his day, and is still I doubt not but a shadow of the perfection to which it is destined to reach, and the great facts it has brought to view, when compared to the discoveries it is destined yet to reveal.

These remarks are applicable to the sciences in general, and to every individual science in particular. Knowledge is but gradually acquired; mind communicates to mind; a master spirit by grave and long continued thought, imparts hints which those who come after ponder, enlarge, elaborate and extend, and transmit as a legacy to subsequent inquirers. How accurately does this delineate, gentlemen, the progress of the profession of medicine? As far back almost as we can trace the history of our race, we meet with the account of disease, and of those who devoted their time and attention to its treatment and cure. Their remedies were simple, and their practice empirical; but some records were kept of it, such as it was, and were appropriated in after times to some good purpose. To reduce this mass of heterogeneous materials, incongruous as many of them must have been, to a system so as to exhibit the phenomena of disease and its treatment, required a mind largely disciplined by close thought, acute perceptions, close observation and discriminating judgment. And such a master mind appeared to undertake the Herculean task. The vigorous, persevering intellect of Hippocrates seemed to be the one suited to set about this great work. I cannot but venerate the good old man, as I see him sitting in the temple of Æsculapius, coyping, analyzing, generalizing and systematizing the tablets there suspended, to put them into a condition to be useful in his day, and to those who should come after. This appears to be the first system of medicine ever compiled, and as such justly deserves much respect; it suggested hints to others, so to collate and arrange what they saw and experienced, as to make them available to other observers. He reached a good old age, dying in his ninety-ninth year. Men of ability and research were not wanting to appropriate what he and others had recorded, and to make large additions of such facts as passed under their immediate notice. But confused theories and a strange desire to harmonize the phenomena of disease with the dogmas of a favorite sect of philosophers, materially retarded the onward progress of medicine for many centuries. Even Galen, although it be admitted that he contributed essentially to the stock of medical knowledge, yet attempted to illustrate every abnormal condition by the principles of the peripatetic philosophy. Hence he very much obscured in his misguided zeal, that which he intended to make plain and intelligible. A learned professor of Leyden said of him, that he thus took a great deal of pains to explain everything into the clouds. The system upon which Galen erected his opinions is certainly very curious; and we can but consider with surprise, that a man learned and intellectual, should in any age, be darkened by such irrational, metaphysical subtleties.

The materia medica of the early ancients was quite as rude as their views of disease. It was drawn from the vegetable kingdom, and being destitute of the knowledge necessary to ascertain the peculiar properties of the articles which composed it, and their adaptation to particular diseases, they could be administered but with uncertain results. Some amusing, if not fabulous accounts of their wonderful effects in the cure of disease have come down to us, such certainly as do not correspond with later observation and experience. Melanpus is said to have cured the daughters of king Proctus of hysteric phrenzy, who imagined themselves transformed into cows, with Hellebore; furnishing possibly the hint for some of the humorous metaphorsoses of the Latin poet Ovid. The history of our profession in these early as well as in later times is entertaining and instructive, and will well compensate for the time devoted to its study.

It has been remarked that the practice of the ancient physicians was empirical; it must have been, and necessarily so, unless our views of correct and rational treatment, require modification or change. Ignorant of almost all those great lights of science, which are requisite to develop an accurate pathology, and the peculiar properties and adaptation of remedial agents, this could not but have been the inevitable result. If we regard every prescription as empirical, which is not based upon a positive knowledge of the particular pathological condition, and of the special lesion of the tissue or organ affected, accompanied with or like positive knowledge that the properties of the agents employed are just suited to remove that abnormal state; and this view seems the true one; and I had almost said the painfully true one; those medical gentlemen, who but a few generations before us, have contributed so largely to the accumulating stock of medical science, whose reputation was so elevated, and usefulness so extended, whose memories we honor and cherish with fond recollections, did but partially possess this positive knowledge of pathology and therapeutics. They knew much of morbid anatomy and analytical pharmacy, and have transmitted legacies of philosophical, scientific, medical truth, which will bless the world to the latest posterity.

Notwithstanding the unprecedented advancement of science and philosophy in our age, and the large attainment in physiology, pathology and therapeutics, the intimate acquaintance cultivated with the structure of the human body, its organs and tissues, its derangements and lesions, truth compels the sad admission, that great uncertainty still attends the healing art; that which we know positively is but limited, and a task devolves upon us sufficient to engage all the energy, perseverance and investigation of ourselves and our profession for ages to come. We cannot in all cases enter the sick room with that confidence of success we desire, and candor necessitates the frequent confession to the anxious inquiry as to the result, that it is not possible to foretell with certainty. This may be affirmed of all things human; imperfection and uncertainty are the indellible impress of humanity; and this, perhaps appropriately, is very commonly regarded as peculiarly applicable to a large portion of medical appliances. It is proper here to inquire, how far the usefulness of our profession is impaired by these uncertainties. It can scarcely be questioned, that it does tend in some measure to diminish it; but all admit the benefits we confer, and consider us, and truly, as a sine qua non, to human relief and comfort.

Those who have cultivated an intimate acquaintance with men have remarked, that the highest happiness of which our nature is susceptible, lies in the hopes and aspirations of the future. No one can doubt this. And this inspiring thought, gentlemen, we seize with an unflinching grasp. We look forward with joyous hope to a period yet to come, perhaps far distant, but that will pretty surely arrive, not of chivalry, not of unbounded wealth, when all shall revel in luxury to satiety; but when truth, absolute truth, and positive scientific knowledge in every department of life shall be attained, and mainly scatter with its clear light, the obscurity and uncertainty which, in a measure, darkens almost every research. We anticipate the period when anatomy, physiology pathology and therapeutics, with the aid of the collateral sciences, shall be so investigated and elaborated, as to demonstrate almost every morbid condition with nearly mathematical certainty. Should these expectations bring upon us the charge of visionary enthusiasm, or that they are the mere hallucinations of a morbid brain, we have, as an irresistible reply, simply to point to the unprecedented advances in all these sciences the last half century, even to the last ten years, to silence all such cavils. Every year new facts are elicited, and distinct progress gained. And why should it not be thus? We have in our ranks, very many of enlarged powers, of indomitable perseverence and research, and who will not stay their investigations short of full attainment. Theology and law furnish bright specimens of learning and talent; and the records of philosophy, science and literature attest, that medicine has furnished a still larger quota. No obstacles, then, can, and no obstacles will, hinder our science from finally reaching that degree of perfection, that intellect and energy is capable of attaining.

Thus far, we have considered only a learned and scientific profession. But, on every side, we find ourselves surrounded by charlatans who are simply pretenders; but by bluster and boasting contrive to gain the confidence of many, and of some, even, whose position and knowledge would seem to present a barrier to such impositions. These are of all grades, but all make the most extraordinary pretensions. One is an electic, and boasts that he selects all that is valuable and true from all systems; another who asserts that heat is life, and cold is death, promises to cure all diseases with capsicum and lobelia. Every variety and form of quackery that the immagination can conceive of, is constantantly met with, and it would be tedious to trace them all through their various grades, till we come down to the low grade of the pill and syrup dealer. And to keep pace with the progressive age, even the spirits are invoked to lend their aid in this work of imposture. These we cheerfully leave to exult in their own ignorance, and to the follies of those who are their willing dupes. But a system of medical frauds, which commenced with Hahneman, in Germany, founded on the false principle that "*similia similibus curantur;*" we can more truthfully say, "*ut similia similibus distrahuntur.*" This imposture, promising so much and accomplishing so little, enlarging and expanding, has reached our shores; and the human mind is so deeply imbued with the propensities of the ancient Athenians, constantly to seek and to receive something new, that many should adopt it can afford no surprise. It presents quite a fascinating garb; assumes to be very genteel, polished, classical, logical, and precisely suited to the most elevated social position, to correspond with the onward progress of the age, and with its delicate little infiniteissimal pellicles, far exceeds the huge nauseous doses of the old scientific school in facility of administration and in practical success. But observation and experience, our standard of medical truth, proves this an empty boast. It is true that most children love sweet things, and some grown up children too; and it is equally true that when the human system is suffering a pathological state, a nothing of a quantity which, when in health, will produce that state, is not an adequate remedy. This imposture must disappear, like multitudes which preceded it, before enlightened reason and sound common sense. The veritable historian, Diedrich Nickerbocker, relates of the redoubtable Governor Van Twiller, the notorious doubter and smoker, that he smoked and doubted, and doubted and smoked, till at last he evaporated in his own smoke. So with pretentious Homœpathy, which is the very sublimation of quackery, some equally truthful historian will yet record that it ultimately vanished in its own complete nothingness and its own utter impotence. The surgical department of our profession engaged the attention of the ancients, and fully keeps pace with our forward progress. Has it not the advantage, in the clear ocular inspection of most cases which fall to its care, over those which are purely medical, and which are oftentimes obscured by indefinite, uncertain and imperfectly developed characteristics? Surgery has certainly attained an enviable position. The vast multitude of preserved lives and limbs, fully demonstrates its high attainments and scientific accuracy.

We aim at a larger amount of knowledge, and we enjoy the positive certainty that a still larger amount of knowledge is within our reach. and will certainly be acquired. In this, every physician shares a personal responsibility. No one, however obscure his position, and however limited his powers, and even young in years, if practically inclined and engaged, but can contribute something to the common stock. It is not true, that he who has spent the greatest number of years in active practice, or prescribed for the greatest number of patients, is necessarily possessed of the largest amount of observation and experience. Oppressive avocations, indolence or indifference may deter him from recording, arranging, analyzing and systematizing the valuable facts that pass before him, so that they are useless to himself and others, But such do not legitimately belong to our fellowship. Even the young man, if a scrutinizing observer, and endowed with acute perceptions, which are indispensable elements of the mind of the physician, by pondering and collating the facts that pass under his view, will in a short time accumulate a very material sum of valuable, practical experience. While life continues, the medical man must inevitably remain a student. He may pass a term of pupilage and graduation, but his education is but then commenced. By applying all his powers and energies under the lucid instruction of his great master and teacher, Nature, as his usefulness and success imperatively require him to do; she will unfold to him her rich, inexhaustible stores of truth; an ample compensation for the most patient, untiring research. "*Natura duce*," "*res maximas consummemus;*" and she ever has, and ever will put to the blush every fanciful, imaginary speculation.

It is stern facts with which we have to deal, and it is stern facts alone we wish to study. Thus progress has been, and inevitably must and will be made. What, gentlemen, are the obligations that devolve upon us in this great matter, so vital to ourselves and the world? You and I have personal responsibilities which we cannot escape if we would, and I trust would not if we could. We occupy a noble position, are, by wise authority, members of a useful and elevated profession; and we owe it to our employers, and also to ourselves, to note carefully every development in every case, and apply to it all our own individual experience, and the recorded experience of those who have gone before us, and that of our cotemporaries. By this, and this alone can we fulfill, and by this we can not fail to fulfill our high calling and destiny. Our city affords peculiar facilities for this good object. Our hospitals, colleges and dispensaries, all of them furnished with medical gentlemen affable, scientific and skillful; inferior to no others in their several departments, and which are cordially open to all inquirers after truth. They furnish the best of opportunities to note disease in all its varieties and modifications, and to test the effects of remedial agents. I say free, for I regard every medical college, hospital and dispensary as free at suitable times and hours to all those in pursuit of those observations and facts which will enlarge and enrich their own minds, and furnish data to enlarge and enrich the minds of others.

The monthly scientific meetings of our Society when we come together fraternally to receive reports, pathological and scientific specimens, and communications, and interchange opinions, views, observations and experiences, will compare most favorably with those of any similar association. We have among our number gentlemen of no ordinary talent, learning, science and skill. We are now regarded as the most active, efficient county organization in our State, which is truly an enviable position. Let us maintain it; let no obstacle interpose to lower this elevated standing. We possess the ability, the means, and I trust the energy and the will, and the love of our profession, thus to sustain our Society: "*Nomen servemus.*"

Our aspirations lead us to aim at a positive and perfect knowledge of disease in all its modifications and varieties, and an equally positive, successful treatment, so as that life may be indefinitely prolonged. But this is not within the scope of human attainment. The unalterable fiat has gone forth, that dust must return to dust. We can only well, honorably and usefully maintain our lot, and bequeath to those who come after us, whatever we have discovered of science or of truth.

